# Outbreak of Tuberculosis and Multidrug-Resistant Tuberculosis, Mbuji-Mayi Central Prison, Democratic Republic of the Congo

**DOI:** 10.3201/eid2411.180769

**Published:** 2018-11

**Authors:** Michel Kaswa Kayomo, Epco Hasker, Muriel Aloni, Léontine Nkuku, Marcel Kazadi, Thierry Kabengele, Dorcas Muteteke, François Kapita, Alphonse Lufulwabo, Ya Diul Mukadi, Jean-Jacques Muyembe-Tamfum, Margareta Ieven, Bouke C. de Jong, Marleen Boelaert

**Affiliations:** Institute of Tropical Medicine, Antwerp, Belgium (M.K. Kayomo, E. Hasker, B.C. de Jong, M. Boelaert);; National Tuberculosis Program, Kinshasa, Democratic Republic of the Congo (M.K. Kayomo, M. Aloni, M. Kazadi, T. Kabengele, D. Muteteke, F. Kapita, A. Lufulwabo);; Institut National de Recherche Biomédicale, Kinshasa (M.K. Kayomo, M. Aloni, L. Nkuku, J.-J. Muyembe-Tamfum);; Université de Kinshasa, Kinshasa (M.K. Kayomo, M. Aloni, J.-J. Muyembe-Tamfum);; US Agency for International Development, Washington, DC, USA (Y.D. Mukadi);; University of Antwerp, Antwerp (M. Ieven)

**Keywords:** tuberculosis, TB, multidrug-resistant, MDR TB, incidence, DRC, prisons, Mbuji-Mayi, Xpert MTB/RIF, Democratic Republic of Congo, DRC, tuberculosis and other mycobacteria, outbreak, bacteria, antimicrobial resistance

## Abstract

After an alert regarding ≈31 tuberculosis (TB) cases, 3 of which were rifampin-resistant TB cases, in Mbuji-Mayi Central Prison, Democratic Republic of the Congo, we conducted an outbreak investigation in January 2015. We analyzed sputum of presumptive TB patients by using the Xpert MTB/RIF assay. We also assessed the *Mycobacterium tuberculosis* isolates’ drug-susceptibility patterns and risk factors for TB infection. Among a prison population of 918 inmates, 29 TB case-patients were already undergoing treatment. We found an additional 475 presumptive TB case-patients and confirmed TB in 170 of them. In March 2015, the prevalence rate of confirmed TB was 21.7% (199/918 inmates). We detected an additional 14 cases of rifampin-resistant TB and initiated treatment in all 14 of these case-patients. Overcrowded living conditions and poor nutrition appeared to be the driving factors behind the high TB incidence in this prison.

Prisons are high-risk environments for tuberculosis (TB) and multidrug-resistant TB (MDR TB) (*1*). Globally, the prevalence of TB in prisons is much higher than in the general population, both in high- and low-income countries (*2*). In sub-Saharan Africa, the spread of TB in prisons has been fueled by the HIV/AIDS epidemic (*3*). Reports from Malawi, Côte d’Ivoire, and Botswana have shown a higher prevalence of smear-positive pulmonary TB in prisons compared with the general population (*4*–*6*). Several factors, such as poor ventilation, HIV infection, overcrowding, malnutrition, lack of sunshine, stress, prolonged incarceration, and inadequate access to care, contribute to the rapid spread and high prevalence of MDR TB in prisons (*2*). 

One of the critical barriers to TB control in prisons is limited access to high-quality TB diagnosis, which is attributable to limited screening, inaccuracy of diagnostic algorithms, and lack of laboratory facilities (*7*,*8*). All those factors are rampant in the Democratic Republic of the Congo (DRC) (population ≈68 million), given its protracted socioeconomic crisis and widespread poverty. Annual TB incidence in DRC is 326 cases/100,000 population (*9*). DRC is a high-burden TB country and has a high incidence of rifampicin-resistant TB (*10*,*11*). Preliminary data from an antimicrobial drug resistance survey in 2018 show a prevalence of rifampicin-resistant TB of 2.2% (95% CI 1.0%–3.5%) among new patients and 16.7% (95% CI 9.6%–23.7%) among previously treated case-patients (M.K. Kayomo, unpub. data). 

The introduction of the Xpert MTB/RIF assay (Cepheid, Sunnyvale, CA, USA) is an important breakthrough in the fight against TB and MDR TB (*12*–*14*). The World Health Organization recommends this assay as a first-line diagnostic test for persons with suspected pulmonary TB who are considered to be at risk for harboring MDR TB bacilli (*15*). At the end of 2013, the DRC National Tuberculosis Program (NTP) progressively introduced the Xpert MTB/RIF technology in the provincial laboratories of DRC, mainly motivated by the lack of data on MDR TB incidence and the extremely long turnaround times associated with conventional methods. In November 2014, the NTP laboratory in Kasai Oriental Province switched from microscopy to the Xpert MTB/RIF for use in Mbuji-Mayi Central Prison because the laboratory had a large stock of cartridges that were due to expire soon. By the end of 2014, the laboratory had confirmed TB in 31 of 57 sputum specimens from prisoners with presumptive TB; this number included, for the first time in the prison records, 3 patients with rifampicin-resistant TB. A total of 72 documented TB cases occurred in the prison that year, almost twice the 2013 figure. 

After being alerted about the situation, the NTP head office in Kinshasa launched an outbreak investigation on January 5, 2015. Two NTP experts (M.K.K. and D.M.) were sent to review the patient histories, ascertain the emergence of TB and rifampicin-resistant TB, and implement infection control measures. We report here the outcomes of this outbreak assessment, including the incidence of TB and MDR TB, the drug-susceptibility patterns of the circulating *Mycobaterium tuberculosis* isolates, and associated risk factors for TB infection.

## Methods

### Ethics

The national health ethics review board of DRC (Comité National d’Ethique de la Santé) gave ethical clearance for this study (document no. 09/CNES/BN/PNMMF/2015). For this report, we only used specimens and data collected in the course of routine patient care and drug-susceptibility surveillance. Data were delinked from any personal identifiers before data analysis and reporting. All persons who had TB or rifampicin-resistant TB diagnosed received the recommended treatment regimen (6 months for TB and 9 months for rifampicin-resistant TB). Patients with rifampicin-resistant TB were isolated at Dipumba General Hospital (Mbuji-Mayi) in a dedicated ward.

### Setting

Mbuji-Mayi, the capital of Kasai Oriental Province (population ≈6.7 million), is located ≈1,000 km east of Kinshasa, the capital of DRC. In 2013, the province had an estimated annual TB incidence of 229 cases/100,000 population (M. Kazadi, unpub. data).

Mbuji-Mayi Central Prison is a medium-security correctional facility built in 1950 with a capacity of 150 inmates. It is surrounded by schools, houses, and government offices. It houses on average 900 inmates (i.e., 6 times its capacity) in 9 cells (7 cells for men, 1 for women, and 1 for juvenile inmates 15–17 years of age).

**Figure 1:**
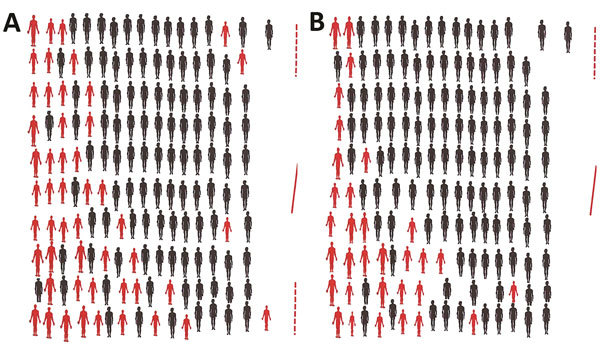
Location of inmates in cells 4 and 7, Mbuji-Mayi Central Prison, Democratic Republic of the Congo, February 2015. A) Cell 4 is 37 m^2^, with 1 door (solid red line) and 2 windows (dashed red lines). B) Cell 7 is 37 m^2^, with 1 door (solid red line) and 1 window (dashed red line). Red figures indicate TB patients. Both cells are in the designated area 2 and are extremely overcrowded, having >163 inmates in each. Each inmate was assigned a space of 0.22 m^2^. Most (60%) TB patients were living in the rear of the cell, which was characterized by poor ventilation and lack of sunshine. This drawing shows the nearly exact localization of inmates during their stay inside the cell; more space is available at the entrance of the cell, which is occupied by the “chief” of the cell. TB, tuberculosis.

The number of prisoners per cell varies from ≈130–160 in the large (36 m^2^) cells to 20–30 in the small (28m^2^) cells, which are also called VIP or first-class cells. On arrival, each prisoner is assigned a fixed spot, which in the regular cells is no larger than ≈0.25 m^2^ (Figure 1). Each cell has >1 window, but prisoners’ clothes and other possessions usually cover these. Inmates receive ≈5 hours of sunshine exposure per day in a courtyard measuring 375 m^2^. They eat with the inmates of the same cell but meet those of other cells during morning sessions, gym, and vocational training. They also have close contact with prison staff, judges, and their own families. The duration of incarceration ranges from 1 month to >15 years. The prison has a clinic, run by 1 medical doctor and 2 healthcare workers.

Located close to the prison is an NTP clinic that conducts direct smear microscopy (no radiology) and can provide TB treatment to prisoners. Prisoners were not routinely screened for TB on entry. The NTP national policy on screening in prisons instructs chest radiograph screening upon entry, followed by smear microscopy if the radiograph results are suggestive. Further screening has to be systematically conducted every 6 months and upon release. However, prison-based TB control measures in DRC are limited in practice because of lack of resources. Until November 2014, only passive case detection for TB based on smear microscopy was implemented in Mbuji-Mayi Central Prison.

### Study Procedures

The outbreak investigation team reached Mbuji-Mayi mid-January 2015 and reviewed all available NTP records as well as the prison admission register and patient files. The team also extracted from the NTP registers data on the TB notification rate in this prison for the 7 years preceding the investigation.

Assisted by provincial-level program staff and prison medical personnel, the team screened all inmates for presumptive TB. A standard form was used to collect data on previous history of TB, symptoms, duration of stay, and location in the cell. Awareness was raised among inmates about the signs and symptoms of TB, and the chief inmate of each cell as well as 10 peer educators, all inmates, were trained to recognize the major symptoms of TB. Inmates with a history of TB or clinical symptoms (e.g., coughing for >2 weeks, fever, night sweats, loss of weight, and hemoptysis) were considered to be presumptive TB patients. These patients were asked to submit 1 early morning sputum sample. Samples were transported to the provincial reference laboratory located 3 km from the prison.

### Laboratory Procedures

#### Xpert MTB/RIF

Fresh sputum samples (without any additive) were transported in standardized containers from the prison to the provincial reference laboratory to be analyzed using the Xpert MTB/RIF assay. We also recorded demographic data of patients and their TB history. A 2-mL aliquot of each sputum sample was processed in Xpert according to standard methods (*12*,*16*). From each specimen showing resistance to rifampicin, an aliquot was preserved in 70% alcohol in a Falcon tube to permit additional molecular testing in Kinshasa and at the Supranational Reference Laboratory (SRL) in Antwerp, Belgium. One aliquot without additive was transported to Kinshasa for culture.

#### MTBDR*sl*

At the Institut National de Recherche Biomédicale in Kinshasa, GenoType MTBDR*sl* (Hain LifeScience GmbH, Nehren, Germany) was performed on specimens showing resistance to rifampicin by Xpert MTB/RIF. Sputum samples were decontaminated with NaOH according to a modified Petroff technique (*17*). The sediment obtained was inoculated on Lowenstein-Jenssen and tested by using MTBDR*sl* according to the manufacturer’s instructions (*18*).

#### Genetic Sequencing

For all available DNA extracts harboring second-line resistance patterns on MTBDR*sl*, sequencing of the *gyrA* and *rrs* genes was performed independently of line probe assay (LPA) results at SRL. The methodology for PCR amplification and sequencing of genes encoding gyrase A and B has been described elsewhere (*19*).

### HIV Screening

The National HIV Program organized a mass HIV screening campaign in the prison, a few days after our arrival in Mbuji-Mayi. Skilled counselors performed pretest group counseling. Posttest counseling was carried out in a private one-on-one setting by the same counselors. Persons who tested positive were referred to the nearby NTP clinic for TB and HIV treatment.

### Data Analysis

All data were double-entered into an Excel 2007 worksheet (Microsoft, Redmond, WA, USA). Both datasets were compared by using Epi Info 7.1.4 (Centers for Disease Control and Prevention, Atlanta, GA, USA); in case of discrepancies, we verified the source document. The primary outcomes of interest for this study were the prevalence rates of TB and rifampicin-resistant TB, computed as the proportion of confirmed TB or rifampicin-resistant TB patients over the total number of prisoners at the time of our visit. These data were later linked with DNA sequencing results from SRL. For this analysis, we divided the prison into 3 areas. Area 1 comprised cells 1–3 (the VIP or first-class cells, where inmates get better conditions in exchange for payment). Area 2 comprised cells 4–7 (the second-class cells). Area 3 comprised cells 8 and 9, the areas for women and juveniles. We mapped the spatial distribution of TB in the cells on a sketch showing the location of each prisoner.

We calculated the body mass index (weight in kilograms/height in meters) as a measure of adult nutritional status and used the cutoffs proposed by Bailey and Ferro-Luzzi (*20*) to define 3 categories of adult malnutrition (severe, moderate, and mild).

We calculated means (including SDs) and medians and ranges for continuous variables. Comparisons between groups were made by using Fisher’s exact test, and 95% CIs were calculated when appropriate.

## Results

### Pulmonary TB

In November 2014, a total of 31 inmates had been found to be positive for TB by Xpert MTB/RIF (4 in area 1 and 25 in area 2). For 2 patients, no information on location was recorded, and they were not present any longer during our outbreak investigation. In January 2015, a total of 918 inmates were housed in Mbuji-Mayi Central Prison, 863 (94.0%) men and 26 (2.8%) women; 29 (3.1%) were juveniles. Most (716/918 [78%]) were pretrial inmates, and only 202 (22.0%) had been sentenced to imprisonment. Area 1 housed 206 inmates (22.4%), area 2 housed 657 inmates (71.6%), and area 3 housed 55 inmates (6.0%). Median age of inmates was 30 years (interquartile range 25–42 years), and 29 inmates were already undergoing TB treatment (all 29 had TB diagnosed after their entry into the prison). Out of the remaining 889 inmates, 45 were absent for various reasons or declined to be screened. 

We clinically examined 844/918 inmates (91.9%) and collected sputum samples of the 475 presumptive TB patients (51.7% [475/918] of inmates). The mean age of inmates with presumptive TB was 32 years (median 31 years), and their mean duration of incarceration was 72 months (median 42 months, range 1–437 months). One Xpert MTB/RIF assay was performed per patient, and 460 valid tests were included in the final analysis, of which 170 were *M. tuberculosis*–positive and rifampin-sensitive and 14 were *M. tuberculosis*–positive and rifampin-resistant. The remaining 276 tests were negative. Thus, by using the Xpert MTB/RIF assay systematically, we raised the total number of TB cases detected during November 2014–March 2015 to 201 (31 initial cases plus 170 additional cases). The overall prevalence rate of TB among the 918 prisoners housed in March 2015 was 21.7% (199/918), including 2 women and 1 juvenile.

### HIV

Most (85.6% [753/879]) inmates agreed to attend a pretest counseling session for HIV (Table 1), but only 539 (71.5%) were tested for HIV because of a shortage in test kits. The overall proportion of inmates with HIV infection was 1.5% (8/539) among inmates tested, 2.6% (5/196) among inmates with bacteriologically confirmed TB, and 0.9% (3/343) inmates without TB (p = 0.12).

**Table 1:** HIV screening results among inmates at Mbuji-Mayi Central Prison, Democratic Republic of the Congo, March 2015

Characteristic	No. (%)
No. inmates housed in the prison	918 (100)
No. inmates who received pretest counseling	879 (95.7)
No. inmates who accepted testing	753 (85.6)
No. inmates tested	539 (71.6)
No. HIV-positive inmates	8 (1.5)
No. inmates with tuberculosis and HIV co-infection	5 (0.9)

### Trend of TB

**Figure 2:**
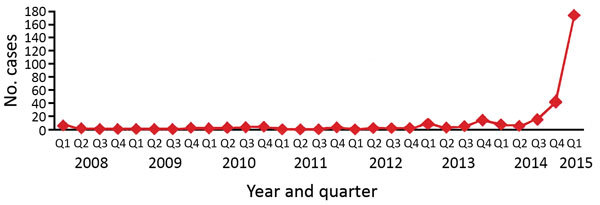
Number of new TB cases registered per quarter, Mbuji-Mayi Central Prison, Democratic Republic of the Congo, 2008–2015. TB cases include bacteriologically positive and clinically diagnosed TB patients. Clinical diagnosis was based on >1 TB-related sign or symptom, a chest radiograph abnormality consistent with TB infection, or both. During 2008–2014, bacteriologic confirmation was based on microscopic results from sputum samples collected through passive case-finding. Xpert MTB/RIF was introduced during the last quarter of 2014. Source: Democratic Republic of Congo National TB Program. Q, quarter; TB, tuberculosis.

From the beginning of 2008 through March 2015, a total of 301 TB patients were registered at Mbuji-Mayi Central Prison. Until the third quarter of 2014, the number of TB patients registered remained relatively stable, with an average of 2 new cases/quarter (range 0–9 cases/quarter). A steep increase was observed from the fourth quarter of 2014 onward (Figure 2), with 212 new TB cases registered (>70% of the caseload since 2008).

### Drug Resistance

By using the Xpert MTB/RIF assay, we found 199 patients to be bacteriologically positive for TB during November 2014–March 2015. Among them, 17 (8.5%) patients (3 initial patients plus 17 additional patients) had rifampicin-resistant TB, which is ≈3 times the expected number of TB-RR cases among new cases according to World Health Organization estimates for DRC (*10*). The overall prevalence of rifampicin-resistant TB in Mbuji Mayi Central Prison in March 2015 was equivalent to 1,852 cases/100,000 inmates. Among the 14 cases documented in March 2015 (Table 2), all but 1 were new cases. For the 3 first detected cases, documented in 2014, the delay between diagnosis to start of treatment was 21 days. This delay decreased to 48 hours for the remaining patients. No second-line drug resistance was identified among any rifampicin-resistant TB patient.

**Table 2:** Selected demographic and clinical characteristics of 14 inmates with diagnosed rifampin-resistant tuberculosis, Mbuji-Mayi Central Prison, Democratic Republic of the Congo, February 2015*

Area	Sex and age, y	History of treatment	Detection date	Treatment started	HIV status	Duration of incarceration, mo
1	M/31	New case	2015 Feb 10	2015 Feb 11	Neg	21
2	M/18	New case	2014 Nov 21	2014 Dec 11	Neg	18
2	M/20	Retreatment	2014 Nov 21	2014 Dec 11	Neg	28
2	M/25	New case	2015 Jan 14	2015 Jan 16	Neg	12
2	M/25	New case	2015 Jan 14	2015 Jan 16	Neg	1
2	M/55	New case	2015 Jan 14	2015 Jan 20	Neg	24
2	M/25	New case	2015 Feb 5	2015 Feb 7	Neg	19
2	M/32	New case	2015 Jan 14	2015 Jan 20	Neg	7
2	M/35	New case	2015 Jan 14	2015 Jan 20	Neg	20
2	M/27	New case	2015 Feb 16	2015 Feb 19	Neg	36
2	M/30	New case	2014 Nov 20	2014 Dec 11	Neg	3
2	M/35	New case	2015 Jan 21	2015 Jan 23	Neg	101
2	M/25	New case	2015 Jan 22	2015 Jan 24	Neg	21
2	M/24	New case	2015 Jan 22	2015 Feb 24	Neg	30

*Drug resistance testing by Xpert MTB/RIF assay. Neg, negative.

Xpert MTB/RIF results showed that all rifampicin-resistant TB was attributable to the absence of probe E. This finding supports the possibility of clonal spread of 1 strain containing a mutation in the *rpoB* 531 codon or, less likely, the 533 codon.

### Risk Factors

#### Overcrowded Living Conditions

Most (60%) TB patients were located in the back of the cell, where ventilation is poor and sunshine is especially lacking (Figure 1). Area 2 (cells 4–7) was the most overcrowded of the 3 areas, housing 657/918 (71.5%) inmates. In cells 4 and 7, the available surface per person was no more than 0.22 m^2^. The frequency of TB and rifampicin-resistant TB increased significantly with the number of inmates per area. Of the 199 confirmed TB patients, 19 resided in area 1, 177 in area 2, and 3 in area 3. The prevalence of confirmed TB was 2.75 times higher in areas 2 and 3 compared with area 1 (25.3% vs. 9.2% [p<0.001]). Out of the 14 rifampicin-resistant TB case-patients identified during January–March 2015, only 1 resided in area 1 (7.1%); the others all resided in area 2.

#### Nutritional Status

Of the 918 inmates, 752 (82%) were screened for nutritional status; of these, 370 (49.2%) were malnourished (body mass index <18.5 kg/m^2^) (Table 3). In the subgroup of 170 confirmed TB patients, 142 (83.5%) were screened for nutritional status; of these, 110 (77.5%) were malnourished. Malnutrition was significantly higher among TB patients than among the other inmates (odds ratio 4.63, 95% CI 3.03–7.08).

**Table 3:** Nutritional status of 752 inmates at Mbuji-Mayi Central Prison, Democratic Republic of the Congo, January 2015*

Nutritional status	No. (%) inmates		
	With TB, n = 142	Without TB, n = 610	Total, N = 752
BMI >18 kg/m^2^	32 (22.5)	350 (57.4)	382 (50.8)
Degree of malnutrition†	110 (77.5)	260 (42.6)	370 (49.2)
Mild	53 (37.3)	95 (15.6)	148 (19.7)
Moderate	27 (19.0)	98 (16.1)	125 (16.6)
Severe	30 (21.1)	67 (11.0)	97 (12.9)

*BMI, body mass index; TB, tuberculosis. †Malnutrition defined as BMI <18 kg/m^2^. Severity of malnutrition: severe, BMI <16.0; moderate, BMI 16.0–16.9; mild, BMI 17.0–18.49.

## Discussion

Our findings provide an account of the high prevalence of TB and drug-resistant TB in a large prison in DRC, where overcrowded living conditions were appalling. The prevalence of TB in this prison was 39 times higher than the estimated 549 cases/100,000 in the general population of DRC (*11*) and is 3.5 times greater than the prevalence reported from prisons in neighboring Zambia (*21*). The TB problem at Mbuji-Mayi Central Prison probably remained undetected for years because of lack of screening and the weak sensitivity of smear microscopy. Some prison studies in sub-Saharan Africa have documented higher rates of TB (*1*,*3*,*7*) in association with a higher HIV prevalence. However, in Mbuji-Mayi Central Prison, we found a relatively low HIV prevalence, although not all consenting prisoners were tested because of a shortage of HIV test kits at the time.

During our investigation, we observed the presence of several risk factors for TB spread in the prison, such as lack of TB screening upon arrival, overcrowding, lack of sunshine, very poor ventilation, and malnutrition (*3*,*22*–*26*). These factors were also documented in other prison TB outbreaks (*3*,*23*,*27*–*32*), but the degree of overcrowding at Mbuji-Mayi Central Prison was a staggering 6 times higher than its capacity. Some prisoners stated that they did not eat for 3 days before our investigation. Malnutrition is a known problem in the Mbuji-Mayi region; the adult malnutrition rate is 45% and >1 million persons require nutritional assistance, according to a 2014 World Food Program report (*33*). Malnutrition tends to amplify TB infection as well as the progress from TB infection to TB disease (*34**–**36*).

One limitation of our study is that no further investigations were conducted to check if the *M. tuberculosis* strains were related, so no hard evidence is available to attribute the high TB and rifampicin-resistant TB rates to in-prison transmission or to a high prevalence among new arrivals. Probably both factors were at work. The preliminary results of a nationwide antimicrobial resistance survey conducted in 2018 (M.K. Kayomo, unpub. data) show that the prevalence of rifampicin-resistant TB in Mbuji-Mayi was 10.4% (95% CI 0.1%–15.6%) among new patients (5 times the national average) and 36.3% (95% CI 18.9%–38.7%) among patients who were previously treated (2 times the national average). The high TB rates in Mbuji-Mayi Central Prison reflect this problem in the community, but in all probability the prison environment acted as an effective amplifier.

As an immediate response measure, the NTP team initiated TB therapy for all confirmed TB case-patients and transferred the rifampicin-resistant TB case-patients to a special isolation ward at the local hospital. We also initiated screening of prisoners upon prison entry. Our findings also led to an acceleration of pending judicial proceedings, which resulted in decongestion of the prison, and the World Food Program intervened to provide supplementary feeding.

However, sustained control of TB among incarcerated populations requires sustained efforts (Table 4). Overcrowding should be avoided, and inmates should have access to better nutrition and more sunlight exposure. Dedicated TB control with adequate diagnostic technology is needed. The active case-finding deployed in Mbuji-Mayi Central Prison using the more sensitive Xpert MTB/RIF assay increased TB case detection by 19-fold. The Xpert MTB/RIF assay offers rapid and accurate diagnostic results from biologic specimens and requires only minimal staff training (*12*–*14*). In prisons, use of this technology is warranted at entry point and thereafter. Passive and active case-finding should be conducted simultaneously and systematically. The high risk for TB in prison settings underscores the urgent need for dedicated TB programs to protect not only the health of prison inmates but also the health of the wider community. However, to avoid outbreaks of MDR TB in similar contexts, living conditions in prisons should be adequate. A hard-learned lesson in prison systems across the world is that safeguarding the basic human rights of these vulnerable populations (including their entitlement to space, food, and health) requires independent monitoring and subsequent action.

**Table 4:** Recommended priority actions to reduce TB prevalence at Mbuji-Mayi Central Prison, Democratic Republic of the Congo*

Action
1. Ensure the screening for TB signs and symptoms of all inmates at the time of prison entry and exit. Continue active and early detection of presumptive TB patients. Raise awareness among the inmates, the prison administration, and the community of the city of Mbuji-Mayi that each cougher should be tested. Confirm presumptive diagnosis by using Xpert MTB/RIF assay. Screen for HIV by using rapid tests.
2. Initiate appropriate treatment of confirmed TB patients within 24 h under strict supervision of healthcare providers.
3. Ensure systematic screening by chest x-ray of the other inmates, the healthcare providers, and the personnel of the prison administration.
4. Feed all inmates adequate and nourishing meals, especially TB and HIV patients undergoing treatment.
5. Ventilate cells.
6. RR-TB patients may be isolated from other inmates at Dipumba General Hospital (Mbuji-Mayi), where the National TB Program was able to obtain a ward for their accommodation. However, the prison administration should find ways to ensure security.
7. Establish compulsory wearing of masks by all TB patients.
8. Establish the compulsory wearing of respirators by all staff entering the prison.
9. Ensure the decongestion of the prison by speeding up judicial proceedings, and increase space by enforcing maximal occupancy levels at the prison.

*TB, tuberculosis.
